# Cytokine Signaling in Splenic Leukocytes from Vaccinated and Non-Vaccinated Chickens after Intravenous Infection with *Salmonella* Enteritidis

**DOI:** 10.1371/journal.pone.0032346

**Published:** 2012-02-24

**Authors:** Marta Matulova, Hana Stepanova, Frantisek Sisak, Hana Havlickova, Marcela Faldynova, Kamila Kyrova, Jiri Volf, Ivan Rychlik

**Affiliations:** Veterinary Research Institute, Brno, Czech Republic; Indian Institute of Science, India

## Abstract

In order to design a new *Salmonella enterica* vaccine, one needs to understand how naive and immune chickens interact differently when exposed to *S. enterica*. In this study we therefore determined the immune response of vaccinated and non-vaccinated chickens after intravenous infection with *Salmonella enterica* serovar Enteritidis (*S.* Enteritidis). Using flow cytometry we showed that 4 days post infection (DPI), counts of CD4 and B-lymphocytes did not change, CD8 and γδ T-lymphocytes decreased and macrophages and heterophils increased in the spleen. When vaccinated and non-vaccinated chickens were compared, only macrophages and heterophils were found in significantly higher counts in the spleens of the non-vaccinated chickens. The non-vaccinated chickens also expressed higher anti-LPS antibodies than the vaccinated chickens. The expression of interleukin (IL)1β, IL6, IL8, IL18, LITAF, IFNγ and iNOS did not exhibit any clear pattern in the cells sorted from the spleens of vaccinated or non-vaccinated chickens. Only IL17 and IL22 showed a differential expression in the CD4 T-lymphocytes of the vaccinated and non-vaccinated chickens at 4 DPI, both being expressed at a higher level in the non-vaccinated chickens. Due to a similar IFNγ expression in the CD4 T-lymphocytes in both the vaccinated and non-vaccinated chickens, and a variable IL17 expression oscillating around IFNγ expression levels, the IL17∶IFNγ ratio in CD4 T-lymphocytes was found to be central for the outcome of the immune response. When IL17 was expressed at higher levels than IFNγ in the non-vaccinated chickens, the Th17 immune response with a higher macrophage and heterophil infiltration in the spleen dominated. However, when the expression of IL17 was lower than that of IFNγ as in the vaccinated chickens, the Th1 response with a higher resistance to *S.* Enteritidis infection dominated.

## Introduction

Non-typhoid salmonellosis together with campylobacteriosis belong among the two most important causes of human gastrointestinal disorders in developed countries. The most important reservoirs of *Salmonella enterica* for humans are found in farm animals, poultry and pigs in particular. Since it is believed that a decrease in the prevalence of *S. enterica* in farm animals will result in a lower incidence of human salmonellosis, measures on how to decrease *S. enterica* prevalence in farm animals are continuously being sought.

One of the possible measures targeted at the decrease of *S. enterica* prevalence in poultry is vaccination. However, in order to design new a *Salmonella* vaccine for chickens with significantly improved performance over the current ones, one needs to understand how both naive and immune chickens interact when infected with *S. enterica*. Earlier studies were focused either on the characterization of cellular infiltrates or cytokine signaling in the infected tissues, mostly in the cecal wall, liver or spleen of chickens orally infected with *Salmonella*. It is therefore known that heterophils form the initial cellular infiltrate [1], [2] followed by the infiltration of macrophages and T-lymphocytes [3]. Recently, significant changes in γδ T-lymphocytes have been described in chickens after *S.* Enteritidis infection [4].

Leukocytes infiltrating the site of infection communicate in a controlled fashion by cytokine release. The cytokines produced after *S. enterica* infection include proinflammatory cytokines and chemotactic chemokines such as IL1β, IL6 or IL8, Th1 cytokines such as IFNγ and Th17 cytokines such as IL17 or IL22 [5]. A lower level of cellular infiltrate and a lower level of cytokine expression were commonly observed in the tissues of chickens that had been vaccinated prior to the infection than in those exposed to the infection for the first time [6]–[8]. However, since cytokine signaling is usually determined by real-time PCR using RNA/cDNA isolated from whole target tissue, information about the contribution of individual cellular subpopulations in chickens is essentially unavailable. And if there were attempts to determine cytokine signaling in particular cell population of chickens, e.g. γδ T-lymphocytes [9], this was performed alone not providing sufficiently general overview on the immunological processes occurring in chickens after *S. enterica* infection.

The whole effort towards understanding the chicken immune response to vaccination and (re)infection is also adversely affected by the fact that although newly-hatched chickens are highly sensitive to *S. enterica*, older, non-vaccinated chickens are quite resistant to the infection [6]. However, if a new vaccine is being tested, this commonly requires a primary vaccination on the day of hatching, revaccination 2–3 weeks later and challenge an additional 2–3 weeks later. Following the above vaccination scheme, the challenge at around 6 weeks of age becomes an issue because this is time when even the non-vaccinated birds are already relatively resistant to oral challenge [6]. This is why in this study we focused on the immune response of vaccinated and non-vaccinated chickens after intravenous infection with *S.* Enteritidis. Using flow cytometry we first characterized the dynamics of leukocyte (macrophages, heterophils, B-lymphocytes, CD8, CD4 and γδ T-lymphocytes) infiltrates in the spleen and in FACS sorted leukocyte subpopulations we next determined Th1 and Th17 cytokine expression. This allowed us to characterize roles of individual leukocyte subpopulations during primary and secondary exposure of chickens to *S.* Enteritidis infection.

## Results

### *S. Enteritidis* challenge

**Figure 1:**
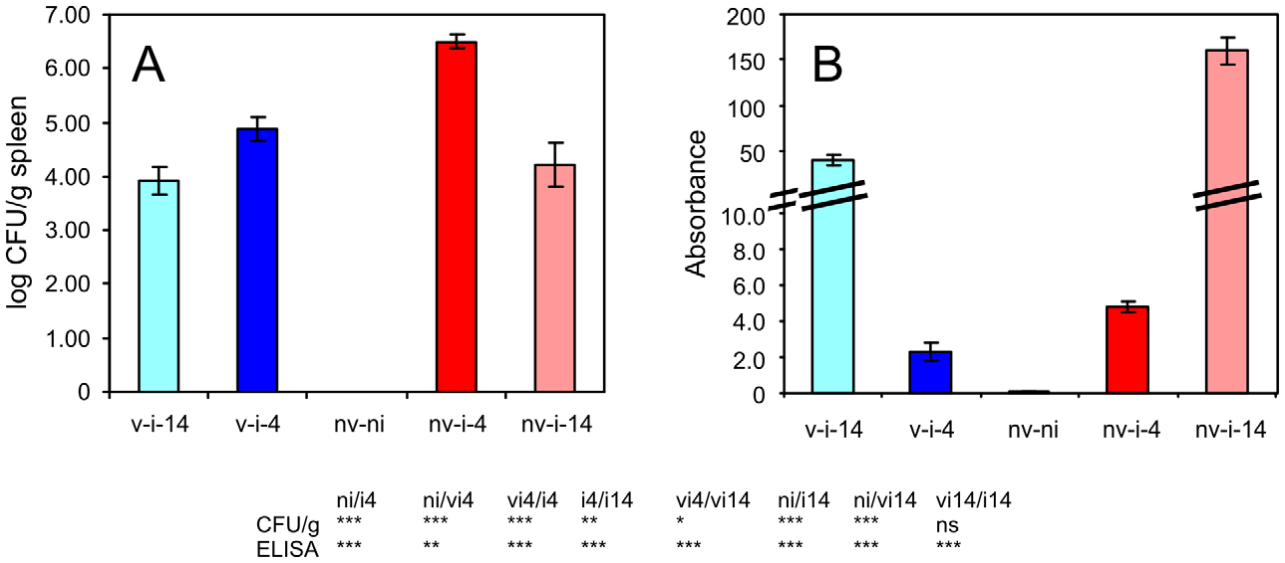
Intravenous infection of chickens with *S.* Enteritidis. Panel A, *S.* Enteritidis counts in the spleens of vaccinated and non-vaccinated chickens, 4 and 14 days after intravenous challenge, respectively. Panel B, serological response to the infection. nv-ni, non-vaccinated and non-infected chickens; v-i-4, vaccinated and infected and 4 days post challenge; nv-i-4, non-vaccinated and infected and 4 days post challenge etc. Table below – t-test comparison of biological relevant groups, ni, non-infected chickens; vi4, vaccinated and infected and 4 days post challenge; ni4, non-vaccinated and infected and 4 days post challenge etc. ns – non-significant difference, * P<0.05, ** P<0.01, *** P<0.001.

Over 10^6^ CFU of *S.* Enteritidis per gram of spleen was recorded in the non-vaccinated chickens at 4 days post infection (DPI). Counts of *S.* Enteritidis more than 10× lower were observed in chickens which had been vaccinated before the challenge. Fourteen days after infection, *S.* Enteritidis counts decreased in the spleens of both vaccinated and non-vaccinated chickens to 10^4^ CFU/g (Fig. 1A).

The intravenous mode of administration of the *S.* Enteritidis used for challenge resulted in a high antibody production. Four days post intravenous infection, higher antibody levels were recorded in the non-vaccinated birds when compared with the vaccinated birds. Fourteen days post infection, a further increase in antibody production was recorded in all infected birds with the non-vaccinated birds exhibiting significantly higher antibody titers than the vaccinated birds (Fig. 1B).

### Cellular infiltrates after i.v. challenge determined by flow cytometry

**Figure 2:**
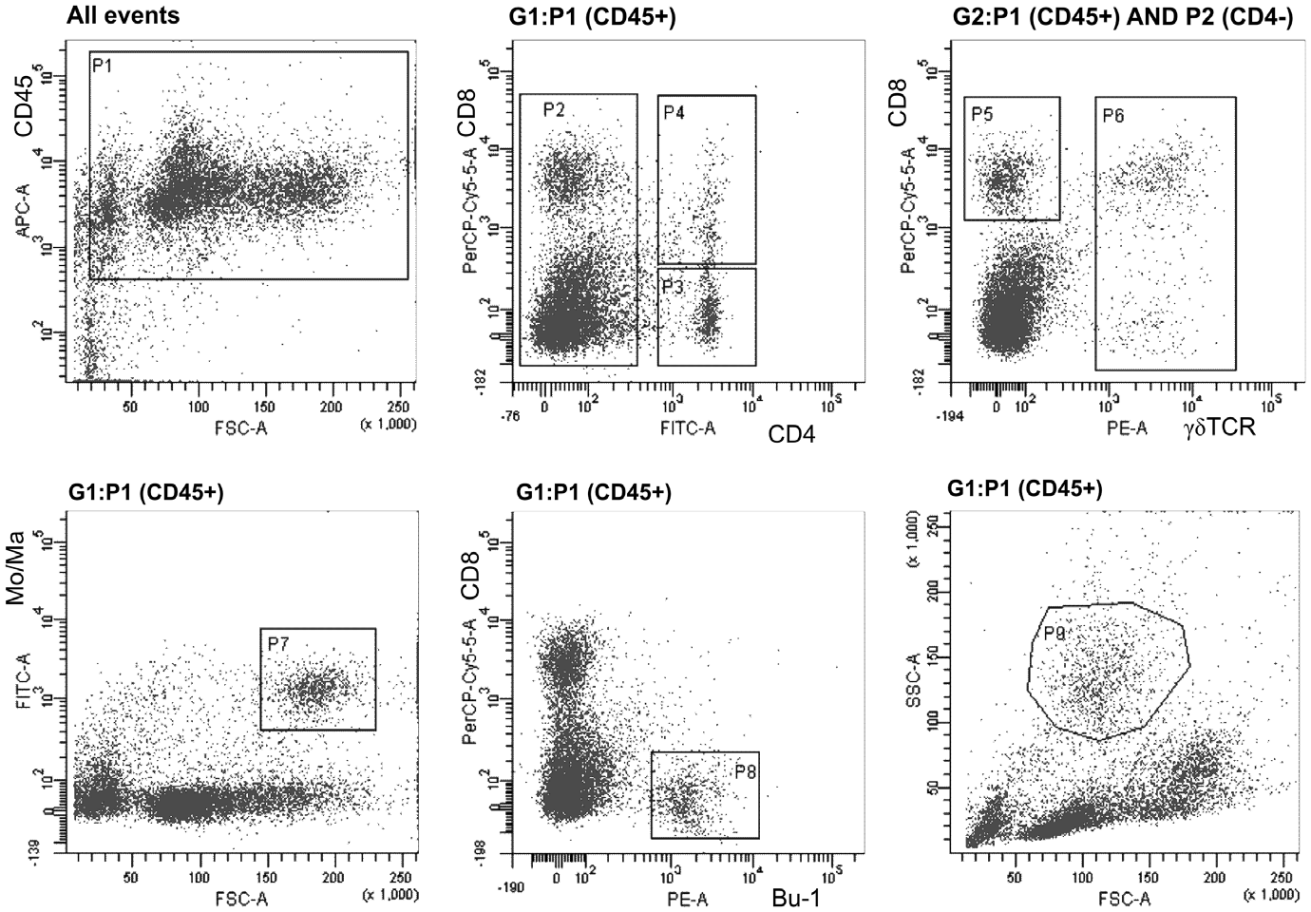
Gating strategy for the characterization of cellular infiltrates in the spleen and for the sorting of leukocyte subpopulations. The leukocytes were gated based on the CD45 expression (P1) and only CD45+ cells were included in the sorting and analyses. Sorted populations were followed: CD4 T-lymphocytes (P4+P3), CD8+ T-lymphocytes (P5), γδ T-lymphocytes (P6), monocytes/macrophages (P7), B-lymphocytes (P8) and heterophils (P9).

**Figure 3:**
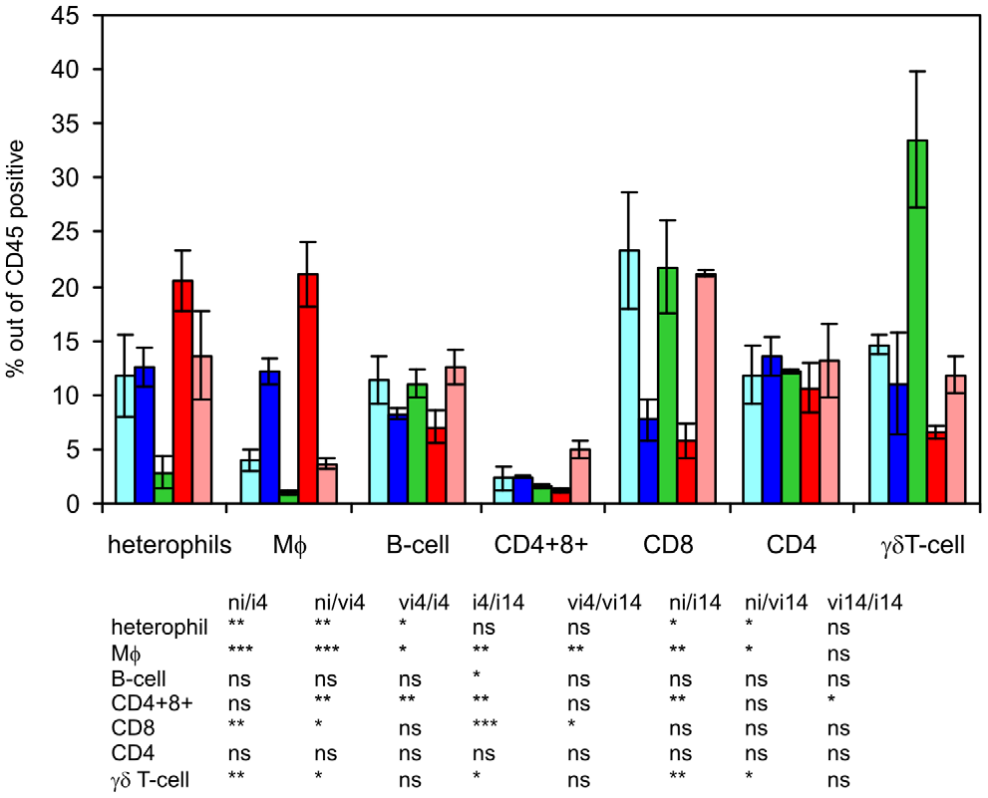
Relative representation of leukocyte subpopulations in the spleens of infected chickens 4 and 14 days post infection. Light blue columns - vaccinated and infected chickens 14 DPI, blue columns vaccinated and infected chickens 4 DPI, green columns – non-infected chickens, red columns non-vaccinated and infected chickens 4 DPI, pink columns - non-vaccinated and infected chickens 14 DPI. Table below – t-test comparison of biologically relevant groups, ni, non-infected chickens; vi4, vaccinated and infected and 4 days post challenge; ni4, non-vaccinated and infected and 4 days post challenge etc. ns – non-significant difference, * P<0.05, ** P<0.01, *** P<0.001.

CD4 lymphocytes, i.e. CD4+ CD8− TCR1−, and B-lymphocytes represented the subpopulation counts which did not change significantly in the spleen of chickens after the challenge (see Figs. 2 and 3).

Double positive CD4 and CD8 lymphocytes, i.e. CD4+ CD8+ TCR1−, were represented at relatively low levels in the spleen. Similar to CD4+ lymphocytes, this subpopulation only weakly responded to the infection at 4 DPI. However, this subpopulation significantly increased in the non-vaccinated chicken at 14 DPI (Fig. 3).

CD8 T-lymphocytes (CD8+ CD4− TCR1−) and γδ T-lymphocytes (TCR1+, CD4− and CD8 either positive or negative) decreased in response to *S.* Enteritidis infection. CD8 T-lymphocytes decreased in counts 4 DPI whilst at 14 DPI their counts returned to those before the challenge. Unlike CD8, γδ T-lymphocytes decreased in number at both time points after the challenge. However, the response characteristics of CD8 and γδ T-lymphocytes did not differ in the vaccinated or non-vaccinated chickens (Fig. 3).

Macrophages and heterophils were the two subpopulations which increased in response to the infection. Monocytes/macrophages increased 4 DPI and decreased nearly to the levels present in the non-infected chickens at 14 DPI. On the other hand, heterophils remained at a high level both at 4 and 14 DPI. Four days post infection, the infiltration of macrophages and heterophils was significantly higher in the non-vaccinated chickens than in vaccinated chickens (Fig. 3).

### Cytokine expression in the spleen, purified leukocytes and sorted cellular subpopulations

**Figure 4:**
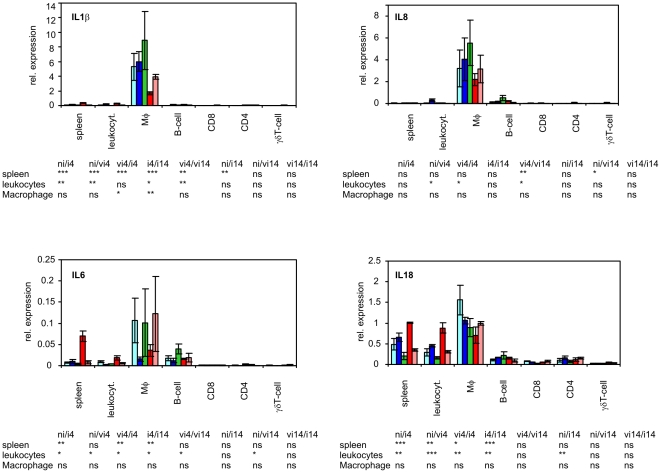
Expression of IL1, IL6, IL8 and IL18 in sorted splenic leukocytes after intravenous *S.* Enteritidis challenge. Light blue columns - vaccinated and infected chickens 14 DPI, blue columns vaccinated and infected chickens 4 DPI, green columns – non-infected chickens, red columns non-vaccinated and infected chickens 4 DPI, pink columns - non-vaccinated and infected chickens 14 DPI. Table below – t-test comparison of biologically relevant groups, ni, non-infected chickens; vi4, vaccinated and infected and 4 days post challenge; ni4, non-vaccinated and infected and 4 days post challenge etc. ns – non-significant difference, * P<0.05, ** P<0.01, *** P<0.001.

There were 3 different groups formed by 9 cytokines tested in this study. The first group comprised of IL1β, IL6, IL8 and IL18 which were produced predominantly by macrophages. Especially for IL1β and IL8, the contribution of any other cell type was marginal. The second group was formed by iNOS and LITAF which were expressed in all the leukocyte subpopulations at similar levels. The last group comprised IFNγ, IL17 and IL22, production of which was dependent on T-lymphocytes (Fig. 4–6).

**Figure 5 pone-0032346-g005:**
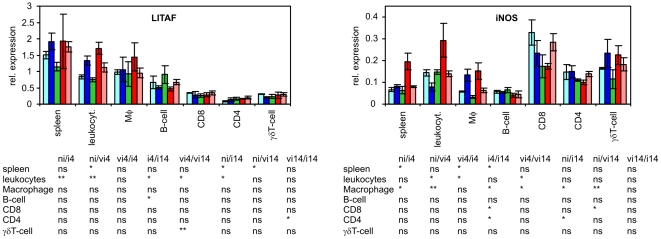
Cytokine expression of LITAF and iNOS in sorted splenic leukocytes after intravenous *S.* Enteritidis challenge. Light blue columns - vaccinated and infected chickens 14 DPI, blue columns vaccinated and infected chickens 4 DPI, green columns – non-infected chickens, red columns non-vaccinated and infected chickens 4 DPI, pink columns - non-vaccinated and infected chickens 14 DPI. Table below – t-test comparison of biologically relevant groups, ni, non-infected chickens; vi4, vaccinated and infected and 4 days post challenge; ni4, non-vaccinated and infected and 4 days post challenge etc. ns – non-significant difference, * P<0.05, ** P<0.01, *** P<0.001.

**Figure 6:**
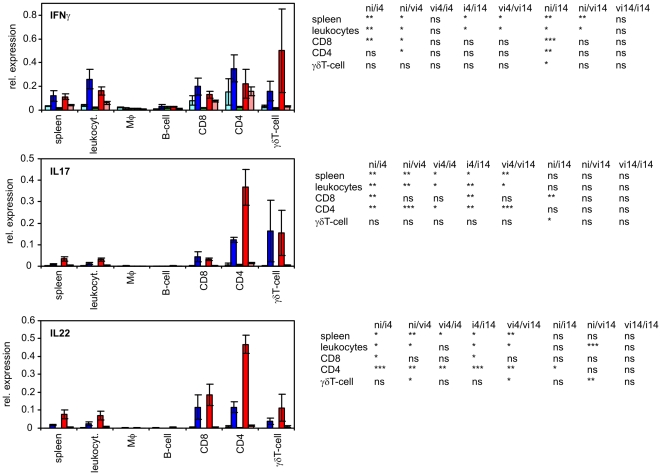
Expression of IFNγ, IL17 and IL22 in sorted splenic leukocytes after intravenous *S.* Enteritidis challenge. Light blue columns - vaccinated and infected chickens 14 DPI, blue columns vaccinated and infected chickens 4 DPI, green columns – non-infected chickens, red columns non-vaccinated and infected chickens 4 DPI, pink columns - non-vaccinated and infected chickens 14 DPI. Table below – t-test comparison of biologically relevant groups, ni, non-infected chickens; vi4, vaccinated and infected and 4 days post challenge; ni4, non-vaccinated and infected & 4 days post challenge etc. ns – non-significant difference, * P<0.05, ** P<0.01, *** P<0.001.

The expression of IL1β, IL6, IL8 and IL18 was dependent mainly on macrophages followed by B-lymphocytes. These cytokines remained expressed at the same level in macrophages after *S.* Enteritidis infection although their expression significantly increased in the spleen. Except for IL1β at 4 DPI, expression profiles of these cytokines did not differ significantly between the vaccinated and non-vaccinated chickens. However, the data for IL1β and IL8 must be taken with care because when comparing their expression in the spleen, total leukocytes and macrophages (considering also the size of macrophage population) it is clear that these two cytokines must have been induced in macrophages during labeling and cell sorting (Fig. 4).

Expression of LITAF and iNOS was not restricted to any leukocyte subpopulation although LITAF was slightly more transcribed in macrophages and B-lymphocytes and iNOS was slightly more expressed in all T-lymphocyte subpopulations. Furthermore, LITAF was not inducible in the sorted leukocytes whilst iNOS was induced in macrophages in response to the infection at 4 DPI. When the vaccinated and non-vaccinated chickens were compared, iNOS was expressed at a significantly higher level in the spleen and the total number of leukocytes of non-vaccinated chickens at 4 DPI. However, there were no significant differences in iNOS expression among any of the sorted subpopulations originating from the vaccinated or non-vaccinated chickens (Fig. 5).

The expression of IL17, IL22 and IFNγ was dependent on the T-lymphocytes and all these cytokines were clearly induced after *S.* Enteritidis challenge. IL17 and IL22 were induced mainly at 4 DPI and decreased at 14 DPI whilst IFNγ remained at an increased transcription rate both at 4 and 14 DPI (Fig. 6). The expression of all these 3 cytokines in CD8 and γδ T-lymphocytes did not significantly differ between the vaccinated and non-vaccinated chickens. Even the expression of IFNγ in CD4 T-lymphocytes did not differ between the vaccinated and non-vaccinated chickens. The only difference in cytokine signaling between the vaccinated and non-vaccinated chicken was the expression of IL17 and IL22 in CD4 T-lymphocytes at 4 DPI. These two cytokines were expressed in significantly higher levels in CD4 T-lymphocytes originating from the non-vaccinated chickens than in those from the vaccinated chickens (Fig. 6).

## Discussion

In this study we were interested in the identification of differences in the immune response between vaccinated and non-vaccinated chickens to *S.* Enteritidis infection. Unlike the majority of previous studies, for the characterization of immune response we used sorted leukocyte subpopulations from the spleens of intravenously infected chickens. Perhaps by adopting the less-frequent, intravenous mode of infection, which resulted in a severe systemic infection in both the vaccinated and non-vaccinated chickens, the differences in cytokine signaling between the vaccinated and non-vaccinated chickens were not great despite the fact that the vaccinated chickens were 10 times more resistant than the non-vaccinated ones, although this was only evident at 4 DPI (Fig. 2A). The i.v. infection itself can be understood as a certain limit of this study. However, a more relevant oral infection of 42-day-old chickens followed by an analysis of splenic subpopulations would likely result in insignificant differences since splenomegaly as an indicator of cellular infiltrates into the spleen is obvious after i.v. infection but is essentially absent after oral infection of 42-day-old chickens [10]. In addition, a similar immune response can be expected also in the spleens of very young chickens infected in hatcheries, in which colonization of the spleen and liver is quite common.

Four days after the infection, an increase in macrophages and heterophils was observed in the spleens of the infected chickens. The changes in the cellular composition in the spleens of the infected chickens were therefore in clear contradiction to the response of the Balb/C mice to the infection with the same *S.* Enteritidis strain [11]. As all the flow cytometry calculations were performed within CD45 positive cells considered as 100%, the increase of macrophages and heterophils should automatically lead to a decrease in all other subpopulations. This may explain the decrease of CD8 and γδ T-lymphocytes. However, as B-lymphocytes and CD4 T-lymphocytes did not change, counts of these two subpopulations had to increase in their absolute counts albeit at lower rate than the macrophages and heterophils. In the case of B-lymphocytes and CD4 T-lymphocytes, the increase in their absolute counts in the spleen due to the infiltration from circulation is as likely as their clonal expansion after antigen stimulation. The clonal expansion of B-lymphocytes is supported also by a high antibody production (Fig. 2B). We also noticed that double positive CD4 and CD8 lymphocytes increased in the spleens of non-vaccinated chickens at 14 DPI. Certain authors proposed that such subpopulation might represent the memory T cells [12]. Their increase at 14 DPI but not at 4 DPI would support this although the absence of their increase in already vaccinated chickens cannot be explained by any current model.

Cytokines with a clear response to the infection and/or a different response in the vaccinated and non-vaccinated chickens included iNOS, IFNγ, IL17 and IL22. The difference in iNOS expression between the vaccinated and non-vaccinated chickens was exhibited more in the spleen and total leukocytes whilst none of the sorted subpopulations exhibited the same expression profile. This apparent contradiction can be explained by a higher infiltration of macrophages into the spleen of the non-vaccinated chickens than in the vaccinated chickens (Fig. 3). This result also shows that the increase in iNOS expression in different tissues after *Salmonella* infection [13]–[15] can be caused both by its induction in macrophages (Fig. 3) and by an influx of leukocytes from circulation to the site of inflammation, as we proposed earlier based on a high iNOS expression in the blood of healthy adult hens [16].

**Figure 7:**
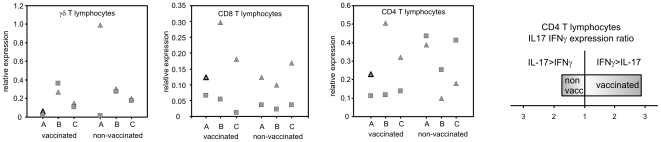
Transcription of IFNγ and IL17 in T-lymphocytes of vaccinated or non-vaccinated chickens 4 days post intravenous challenge. A, B, C - individual chickens vaccinated or non-vaccinated chickens. The most right panel shows polarization towards IFNγ or IL17 transcription ratio in CD4 T-lymphocytes of vaccinated and non-vaccinated chickens, respectively. Squares, transcription of IL17 and triangles, transcription of IFNγ.

The only significantly different response in any of the sorted leukocytes of the vaccinated or non-vaccinated chickens was the expression of two cytokines characteristic of the Th17 immune response, namely IL17 and IL22. For IL17 in CD4 T-lymphocytes we noticed that its expression levels oscillated around the expression levels of IFNγ, a central cytokine of the Th1 branch of the immune response. In the CD4 T-lymphocyte populations from each of the non-vaccinated chickens, the expression of IL17 was always higher than the expression of IFNγ whilst in the CD4 T-lymphocyte populations from each of the vaccinated chickens, the expression of IL17 always dropped below the expression of IFNγ (Fig. 7). At 4 DPI, CD4 T-lymphocytes of the vaccinated chickens therefore produced nearly 3 times more IFNγ than IL17 while CD4 T-lymphocytes from the non-vaccinated chickens produced 1.7× more IL17 than IFNγ. Nothing like this was observed in the CD8 or γδ T-lymphocytes which also induced IFNγ, IL17 and IL22 expression in response to *S.* Enteritidis infection (Figs. 4 and 5). The Th1 immune response with IFNγ as its central cytokine is generally considered as the most important for resistance to *Salmonella* infection and consistent with this, there were lower counts of *S.* Enteritidis in the spleens of vaccinated chickens (Fig. 2A). On the other hand, the characteristics of IL17 signaling and the Th17 branch of immune response include inflammation and leukocyte infiltration. This is in agreement with higher heterophil counts, higher macrophage counts and also higher IL17, IL22 and iNOS expression observed in the spleen of the non-vaccinated chickens (Figs. 3 and 6).

The fact that the IFNγ∶IL17 ratio and that the Th1 branch of immune response is central for resistance to *S.* Enteritidis infection comes also from data at 14 DPI. At this time point, the expression of IL17 and IL22 dropped significantly when compared with 4 DPI and the differences in their expression between CD4 T-lymphocytes from the vaccinated and non-vaccinated chickens disappeared. As the expression of IFNγ did not decline that rapidly, the IFNγ∶IL17 ratio increased to values between 12 and 35 in the CD4 T-lymphocytes from the non-vaccinated and vaccinated chickens, respectively. In both the cases, this polarized the immune response towards a Th1 response and increased the resistance to *S.* Enteritidis infection as documented by the decrease in *S.* Enteritidis counts in the spleen and a reduction in macrophage infiltration of the spleen in both the vaccinated and non-vaccinated chickens. These results are also in total agreement with previous experimental data [7], [15]. Although the results presented in this study were obtained with a rather small number of chickens, in conclusion, we have shown that i) the vaccinated chickens responded to *S.* Enteritidis infection by the Th1 branch of immune response ii) the non-vaccinated chickens responded by the Th17 branch of immune response at 4 DPI, however, at 14 DPI they re-oriented their immune response towards the Th1 branch, iii) the only cellular subpopulation controlling the polarization was formed by CD4 T-lymphocytes and iv) the polarization in CD4 T-lymphocytes was achieved by the regulation in expression of IL17 and IFNγ. Finally, it would be interesting to compare the IFNγ∶IL17 ratio in chickens vaccinated with different live, attenuated *Salmonella* vaccines, both to confirm or exclude whether the observed phenomenon is specific for the vaccination with the SPI1 mutant or whether it is common to all attenuated vaccine strains. In the latter case it would be interesting to correlate the IFNγ∶IL17 ratio with a real protection determined by bacterial counts in the spleen of vaccinated and non-vaccinated chickens after the challenge.

## Materials and Methods

### Ethics Statement

The handling of animals in the study was performed in accordance with current Czech legislation (Animal protection and welfare Act No. 246/1992 Coll. of the Government of the Czech Republic). The specific experiments were approved by the Ethic Committee of the Veterinary Research Institute (permit number 48/2010) followed the Committee for Animal Welfare of the Ministry of Agriculture of the Czech Republic (permit number MZe 1226).

### Bacterial strain and chicken line

*S.* Enteritidis strain 147, a clone spontaneously resistant to nalidixic acid, was used in this study. The construction of isogenic mutant with completely removed Salmonella Pathogenicity Island 1 (SPI-1) used as a live attenuated vaccine for the vaccination of egg laying ISA Brown chickens (Hendrix Genetics, Boxmeer, Netherlands) was described earlier [1]. Immediately after transport, 3 chickens were sacrificed straight away and proved *Salmonella* negative by culture.

### Vaccination and infection

An eighteen-hour-old culture of the SPI-1 mutant grown statically at 37°C in LB broth was used for oral vaccination of 6 one-day-old chickens. The chickens were vaccinated using an oral gavage into the crop with 10^6^ CFU in 0.1 ml of inoculum and revaccinated with the same amount of vaccine on day 21 of life.

The challenge strain was prepared by growth in LB broth at 37°C for 18 hours, pelleting bacteria at 10 000 g for 1 min and resuspending the pellet in the same volume of PBS as was the original volume of LB broth. Three weeks after the revaccination, i.e. on day 42 of chicken's life, the chickens were infected intravenously into the wing vein with 10^7^ CFU in 0.1 ml of wild type *S.* Enteritidis. In addition to the vaccinated chickens, 6 non-vaccinated chickens were intravenously challenged on day 42 of life. Finally 3 non-vaccinated and non-infected chickens sacrificed on day 46 of life were used as controls.

Intravenously infected chickens were sacrificed 4 and 14 days post infection (DPI). At the end of the experiment, blood from each bird was collected for serological tests. During necropsy, approx. 100 mg of spleen was taken for bacteriological analysis, 30 mg was taken into RNALater (Qiagen) for subsequent RNA purification and the rest of the spleen was used for the isolation of splenic leukocytes.

### Cell sorting by flow cytometry

The spleens were collected into ice cold RPMI 1640 medium (Sigma) and during all additional steps the cells were washed or kept on ice. The cell suspensions were prepared by pressing the tissue through a fine nylon mesh. The erythrocytes were removed by cold hemolytic solution (8.26 g of NH_4_Cl, 1 g of KHCO_3_ and 0.037 g of EDTA per liter of distilled water) and the cells were washed twice in 30 ml of cold PBS. After the last washing step, the splenic leukocytes were resuspended in PBS and a small aliquot (2×10^6^ cells) was transferred into 500 µl of Tri Reagent (MRC) to purify the RNA from the total leukocytes. The remaining leukocytes were used for surface marker staining. In total 4×10^7^ of cells were stained in each sample. The first panel of primary antibodies (all Southern Biotech, Alabama, USA) consisted of anti-CD45:APC (clone LT40), anti-CD4:FITC (clone CT-4), anti-CD8α:SPRD (clone CT-8) and anti-TCR1:PE (clone TCR-1). The second panel of antibodies consisted of anti-CD45:APC (clone LT40), anti-monocyte/macrophage:FITC (clone KUL01) and anti-Bu-1:PE (clone AV20). A mouse IgG1 isotype control for each fluorochrome was also used. After 20 min of incubation and subsequent washing in PBS, the cells were subjected to sorting using a FACSAria instrument (BD Biosciences) with a 4 channel sorter. The cells were collected in PBS containing 20% of bovine serum, pelleted by centrifugation and lysed in 500 µl of Tri Reagent. A small aliquot from each sample was left for purity analysis. The purity of sorted populations was: CD8+, 96.7±1.4 (mean%±SD); CD4+, 94.1±2.1; γδTCR+, 93.5±2.6; B-lymphocytes 92.4±3.1; Monocytes/Macrophages, 89.9±3.0. Heterophils were sorted as well, however this population lost its typical FSC/SSC properties and purity of this population was under 60%. Because of this, heterophils were not included in the real-time PCR quantification of the cytokine response. The relative representation of each population was analyzed using FACSDiva software (BD Biosciences) with only CD45+ positive cells included in the analysis. A general gating strategy is shown in Fig. 2.

### RNA purification and reverse transcription

Fifty µl of bromoanisol was added to the sorted cells in 500 µl of Tri Reagent and the samples were vigorously shaken for 10 s. The samples were centrifuged at 4°C for 10 min at 10 000 g, 500 µl of the upper aqueous phase was collected and mixed with an equal volume of 70% ethanol and this mixture was applied to RNeasy purification columns (Qiagen). Washing and RNA elution was performed exactly as recommended by the manufacturer. The concentration and purity of RNA was determined spectrophotometrically (Nanodrop, Agilent) and the RNA was immediately reverse transcribed into cDNA using MuMLV reverse transcriptase (Invitrogen) and oligo dT primers. After reverse transcription, the cDNA was diluted 10 times with sterile water and saved at −20°C prior real-time PCR.

### Real-time PCR

Real-time PCR was performed in 3 µl volumes in 384-well microplates using QuantiTect SYBR Green PCR Master Mix (Qiagen) and a Nanodrop pipetting station from Inovadyne for PCR mix dispensing. Amplification of PCR products and signal detection were performed using a LightCycler II (Roche) with an initial denaturation at 95°C for 15 min followed by 40 cycles of 95°C for 20 s, 60°C for 30 s and 72°C for 30 s. Each sample was subjected to real-time PCR in triplicate and the mean values of the triplicates were used for subsequent analysis. The Ct values of genes of interest were normalized (ΔCt) to an average Ct value of three house-keeping genes (GAPDH, TBP and UB) and the relative expression of each gene of interest was calculated as 2^−ΔCt^. These expression levels were used for data analysis and are presented in the figures as average ± SD. All the primers sequences have been described earlier [13].

### ELISA detection of anti-*Salmonella* LPS antibodies

A commercial FLOCKSCREEN™ *Salmonella* Enteritidis Antibody ELISA kit (x-OvO Limited) was used for the detection of anti-LPS serum antibodies. ELISA was performed exactly as recommended by the manufacturer except for the fact that the sera were diluted from 1∶10 up to 1∶4000 using sample dilution buffer to reach the absorbance which could be measured by spectrophotometer, i.e. ranging from 0.2 to 1.8. Real absorbance was then calculated knowing the read absorbance and particular dilution.

### Statistics

The data were analyzed by both parametric (t-test) and non-parametric tests (Mann-Whitney U test) using Prism Graph Pad Software (La Jolla, USA). Although the results of all statistical tests were very similar (though not identical), the results of the t-test are presented in the results section and in all the figures. In addition, we show the statistics only for biologically relevant data - for example, we do not show the results of the statistical comparison between vaccinated&infected chickens 4 DPI and non-vaccinated&infected chickens 14 DPI, etc. Similarly, since the expression of some of the cytokines was clearly associated with particular cellular subpopulations, we only present the statistical results for the appropriate subpopulations.
